# Testicular Infarction and Rupture After Blunt Trauma — Use of Diagnostic Ultrasound

**DOI:** 10.1100/tsw.2004.101

**Published:** 2004-06-14

**Authors:** Alistair Pace, Christopher Powell

**Affiliations:** ^1^ Department of Trauma and Orthopaedics, Royal Berkshire Hospital, Craven Road, Reading RG1 5AN, UK; ^2^ Consultant Urologist, Department of Urology, Royal Berkshire Hospital, Craven Road, Reading RG1 5AN, UK

**Keywords:** testicular trauma, testicular infarction, ultrasound

## Abstract

We report the case of a 23-year-old male who suffered localised testicular infarction and rupture following blunt trauma. This pathology is rare after blunt trauma and has not been previously described in literature. The appearance on ultrasound resembled malignancy, necessitating orchidectomy. An overview of the pathology of testicular trauma as well as its management is given with particular emphasis on the use diagnostic ultrasound in testicular trauma.

## HISTORY

A 23-year-old, British healthy male presented to the urology department after having sustained a direct kick to his scrotum 2.5 months previously. He complained of pain, swelling, and bruising in his left hemiscrotum.

## EXAMINATION

The left hemiscrotum appeared swollen and there was bruising extending from the scrotum to the thigh. Palpation revealed thickening of the scrotal skin and a tender 1-cm mass in the upper pole of the left testis. The rest of the testis had an abnormal consistency compared to the normal side. The epididymis felt normal. The right hemiscrotum and penis were normal and the inguinal lymph nodes could not be palpated.

## INVESTIGATIONS

An ultrasound examination revealed a hypoechoic, well-defined subcapsular focus 8.4 × 12 mm in size in the upper pole of the left testis (Fig. l). The rest of the testis had an abnormal echoic appearance on

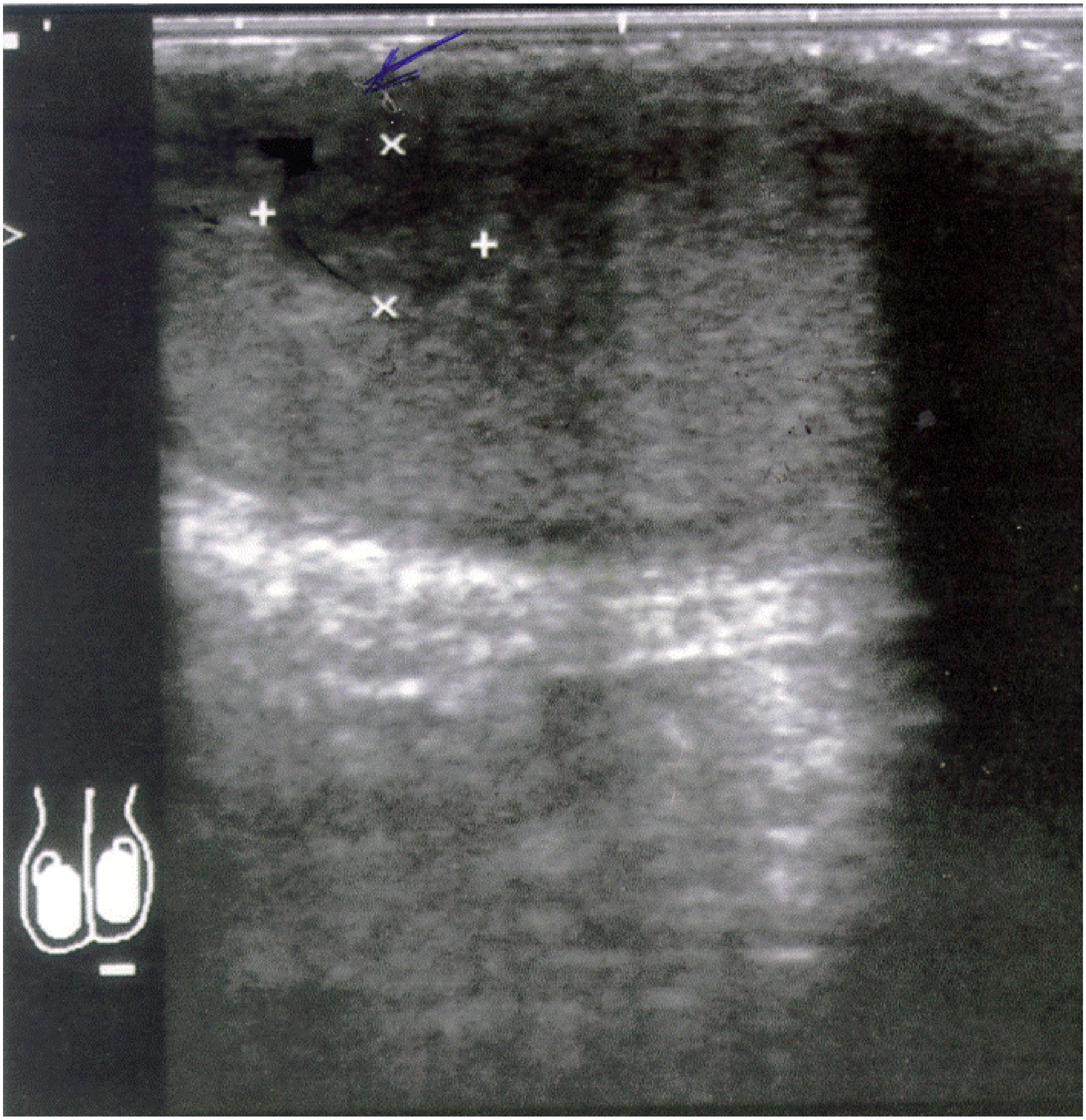

**FIGURE 1**. Ultrasound image showing hypoechoic lesion in the upper pole of the left testis emphasising the difficulty in distinguishing a malignant lesion from testicular infarction. The ruptured tunica albuginea may be identified near the lesion.

ultrasound when compared to the right testis. The blood tests including full blood count, urea, creatinine, and electrolytes as well as alpha feto-protein (A.F.P) and beta human chorionic gonadotrophin (H.C.G.) tumour markers were within normal limits. A testicular tumour could not be excluded and as the testes felt abnormal and appeared suspicious on ultrasound, a biopsy of the testis was felt to be unnecessary and a radical left orchidectomy was performed.

## PATHOLOGY

The macroscopic appearance of the specimen revealed a loculated hydrocoele. At one pole of the testis, there was a haemorrhagic area surrounded by a yellow rim measuring 8 mm across. The haemorrhagic area did not involve the tunica, but there was rupture of the tunica albuginea at the site. The rest of the testis appeared normal. The microscopic examination of the specimen revealed areas of coagulative necrosis and infarction surrounded by an inflammatory reaction including foreign body type granulomatous reaction. Fibrosis and lipofuscin deposits were identified. No malignancy was identified. There were no other areas of necrosis in the testis (Fig. 2).

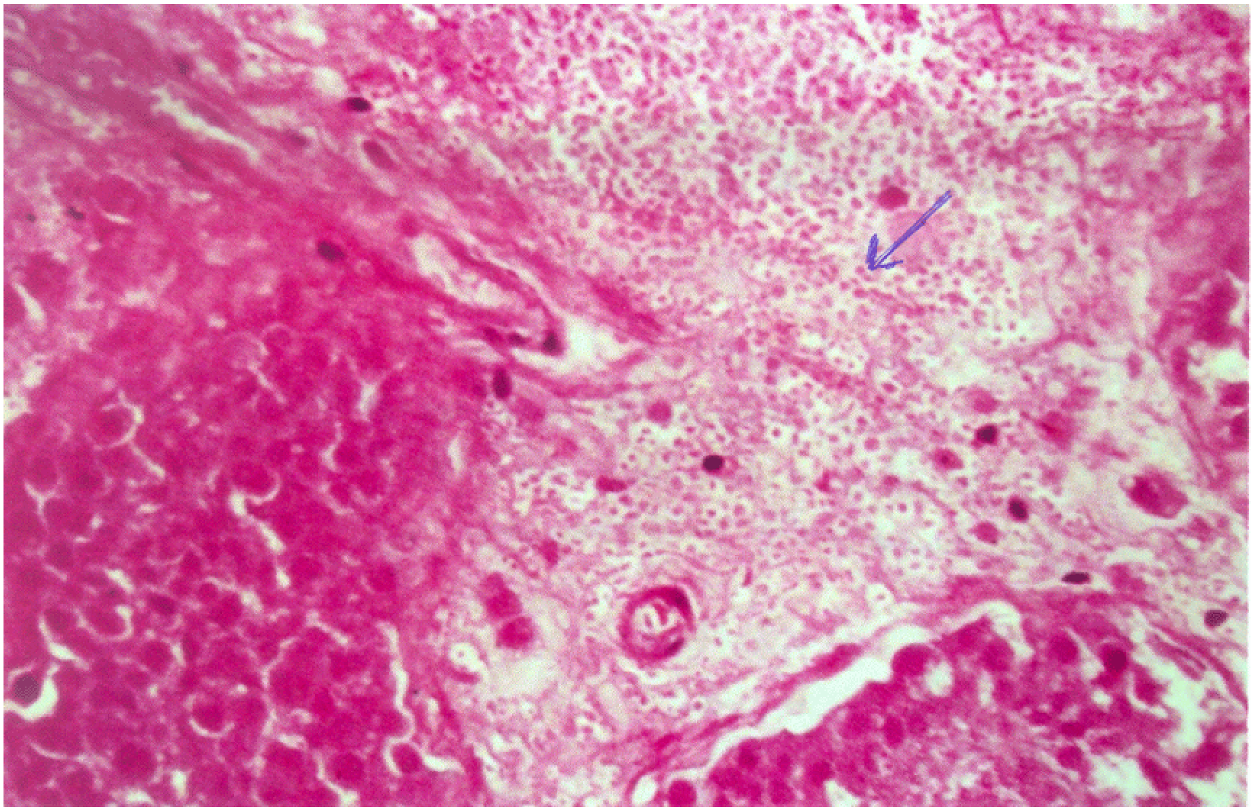

**FIGURE 2**. Histopathology micrograph showing areas of testicular infarction surrounded by an inflammatory reaction. Stain used: H&E Magnification: × 200.

## DISCUSSION

Scrotal trauma has increased in frequency in the last years[1]. Trauma can cause contusions, haematocoeles, rupture of the tunica albuginea, testicular dislocation, intratesticular haematoma, spermatic cord injury, and epididymal haematomas. A large number of testicular ruptures are documented as haematocoeles and are thus missed[2]. As a result, these ruptures are not treated and this leads to atrophy, loss of spermatogenesis and hormonal function, as well as psychological problems[3]. Our patient developed segmental testicular infarction, a condition usually occurring in polycythaemia, sickle cell disease, and hypersensitivity angitis. In this case, the predisposing factor was testicular trauma causing infarction and necrosis with swelling and rupture. This is a very rare consequence of trauma.

The mobility of the testis due to the cremasteric muscle contraction as well as the thick tunica albuginea are responsible for its infrequent injury in multiple injured patients. Direct blows with impingement of the testis against the symphysis pubis may result in testis rupture however [4]. The tear in the tunica albuginea allows the seminiferous tubules to extrude into the tunica vaginalis sac and a haematocoele may form. Moreover, a pole of the testis or the complete testis may be disrupted or shattered[5].

Various studies conclude that exploration with evacuation of blood clots in the tunica vaginalis and repair of testicular rupture increases testis salvage rates; decreases disability, hospital stay, and morbidity; and causes faster return of patients to normal activity when compared to patients managed conservatively. Moreover, in patients managed conservatively, there was increased secondary infection of the haematocoele or the injured testis. Subsequently, necrosis may occur with secondary infection and the pressure effect of the tense haematocoele can cause atrophy of the testis. Conservative management in cases of contusion or rupture of the testis has resulted in a 45% failure rate and 45% orchidectomy rate. This is compared to a 9% orchidectomy rate in patients with testicular trauma who undergo early exploration and repair[1].

Real-time high-resolution ultrasound of the scrotum is an important tool in the management of blunt scrotal injury. Parenchymal heterogenicity or loss of the normal interface between the testis and the tunica albuginea are the hallmarks of testicular trauma[6]. Intratesticular lucencies may represent haematomas, extruded testicular parenchyma, or fragmented testis. Ultrasound may help in assessing blunt testicular trauma by confirming or excluding rupture and torsion and differentiating a soft tissue haematoma from a haematocoele. Doppler ultrasound is useful in investigating the vascular status of the testis. Blood flow to the testis indicates that the vascular pedicle is intact and torsion has not occurred. It is a useful radiological tool but a normal flow may still require exploration if clinically indicated[7,8].

The value of ultrasound in the role of testicular trauma is doubtful according to some authors. Cass states that since exploration provides superior results to conservative management, the role of ultrasound is secondary[2]. On the other hand, Micallef states that physical examination together with ultrasound diagnosis is a highly accurate, readily available noninvasive investigation causing minimal discomfort. It is especially valuable when swelling and distortion due to trauma make examination difficult[4].

Ultrasound examination, however, cannot distinguish between benign and malignant lesions of the testis and in most cases requires exploration of the dubious testis[9]. In our case, the ultrasound examination was inappropiately interpreted and the hypoechoic lesion and abnormal appearance of the rest of the testis was misinterpreted as a malignant lesion resulting in orchidectomy. Patients with testicular tumour may first seek medical advice after trauma and inspite of the ultrasound investigation revealing an avascular rapidly enlarging cystic swelling in a traumatic haematoma and a vascular, slowly increasing mass in a tumour, exploration and perioperative frozen section of the suspicious lesion is required[10].

Table 1 illustrates the advantages and disadvantages of diagnostic ultrasound in blunt scrotal trauma compared to operative exploration. The management strategy adopted in our case did not follow these lines and retrospectively a biopsy or frozen section may have helped in avoiding an orchidectomy.

**TABLE 1**

| Advantages | Disadvantages |
|---|---|
| **Diagnostic scrotal ultrasound** | |
| Accurate | Needs experienced radiologist |
| Available | Difficult to assess perfusion |
| Noninvasive | False negatives; all patients with serious scrotal injury should undergo scrotal exploration unless ultrasound unequivocally shows that the testes are normal. |
| Rapid and reliable | |
| Minimal discomfort | |
| Allows the follow-up of nonoperatively treated cases to resolution | |
| Distinguishes testicular from extratesticular pathology and detects unsuspected pathology | |
| **Operative exploration** | |
| Definite visualisation of injured testis; no false negatives | Complications and risks of operation; general anaesthetic, bleeding, wound infection, further damage |
